# Super-compact treatment with a high dose of moxifloxacin in patients with drug-resistant tuberculosis and its resistance mechanisms

**DOI:** 10.3892/etm.2015.2230

**Published:** 2015-01-29

**Authors:** QINGJIANG WANG, CHUNXIAO ZHANG, JINHUI GUO, JIAN HUANG, XIUE XI, LIGONG ZHANG, XIUQIN CUI

**Affiliations:** 1 Department of Tuberculosis, The First Affiliated Hospital of Xinxiang Medical University, Weihui, Henan 453100, P.R. China; 2 Department of Pharmaceutics, The First Affiliated Hospital of Xinxiang Medical University, Weihui, Henan 453100, P.R. China

**Keywords:** moxifloxacin, multidrug-resistant, tuberculosis, curative effect

## Abstract

The aim of this study was to investigate the curative effect and resistance mechanisms of high-dose moxifloxacin in the short-term treatment of multidrug-resistant tuberculosis. A total of 92 patients with multidrug-resistant tuberculosis were randomly selected and divided into groups A and B (n=46 per group). The two groups received moxifloxacin treatment with the same dose in total. Group A received a short course of treatment with moxifloxacin (0.6 g/day for 6 months), whereas group B received normal moxifloxacin treatment (0.4 g/day for 9 months). Sputum negative conversion, foci absorption, cavity closure and adverse reactions in the two groups were observed, and the drug resistance mechanism of tuberculosis to moxifloxacin treatment was investigated. Following the treatment, the curative rate of group A was 82.61%, and the curative rate of group B was 84.78%; there was no statistically significant difference between the two groups (P>0.05). The rates of sputum negative conversion, foci absorption and cavity closure were not significantly different between the two groups (P>0.05). However, the rates of reduction in peripheral white blood cell counts, liver function damage and adverse reactions, including symptoms affecting the gastrointestinal and nervous systems, were significantly lower in group A than in group B (P<0.05). The expression levels of the antigen-presenting functional molecules CD80 and CD40 on the surfaces of mononuclear cells were higher in group A than in group B (P<0.05), whereas the difference in HLA-DR expression between groups A and B was not significant (P>0.05). In conclusion, short-term treatment with a high dose of moxifloxacin is effective for multidrug-resistant tuberculosis, and its advantages are a reduction in the incidence of drug-associated adverse reactions and a lack of drug resistance.

## Introduction

Multidrug-resistant pulmonary tuberculosis (MDR-PTB) refers to tuberculosis that is resistant to at least two major anti-tuberculosis drugs. MDR-PTB usually cannot be treated effectively by the use of normal drugs and procedures (1). Currently, according to the literature, the fluoroquinolone drug levofloxacin is frequently recommended for use in the treatment of MDR-PTB, and has been widely investigated in clinical trials (2,3). Moxifloxacin is a relatively new synthetic fluoroquinolone antibacterial drug; it has several advantages including high bioavailability, a good curative effect and minimal adverse effects. The newly added methoxy group at position 8 of moxifloxacin further increases its antibacterial activity, and the antibacterial spectrum of moxifloxacin is wider than that of levofloxacin (4). In addition, moxifloxacin has previously been used in the treatment of MDR-PTB, the majority of previous studies have reported only on the efficacy of moxifloxacin in the treatment of multidrug-resistant tuberculosis(2,4). The mechanism of moxifloxacin on multidrug-resistant tuberculosis has received less investigation.

In the present study, the curative effect of a compacted treatment schedule of moxifloxacin was investigated in 92 patients with multidrug-resistant tuberculosis, randomly selected from January 2011 and March 2013. Forty-six of the patients received a high dose of moxifloxacin in a short-term treatment, and were compared with the remaining 46 patients who received moxifloxacin at the usual dosage and treatment duration. The mechanism of multidrug-resistance in the patients was also investigated.

## Subjects and methods

### Subjects

A total of 92 patients with MDR-PTB, which was confirmed by tuberculosis culture test from January 2011 to March 2013, were randomly selected. The patients were randomly divided into two groups, group A (n=46) and group B (n=46). The inclusion criteria are as follows: i) received anti-tuberculosis drug treatment for 1 year and had positive smear test results; ii) resistant to at least two anti-tuberculosis drugs after improved Lowenstein-Jensen medium susceptibility testing; iii) no history of allergy to the drugs used in this study; and iv) tuberculosis lesion detected in the lung by computed tomography or magnetic resonance imaging examination. Patients with liver and kidney dysfunction, psychology or other basic diseases were excluded. In group A, there were 34 male and 12 female patients, with a disease course of 1–24 years (mean, 11.25±7.56 years), with cavity closure in 35 cases (48 cavities in total). In group B, there were 32 male and 14 female patients, with a disease course of 2–23 years (mean, 13.15±8.10 years), with cavity closure in 36 cases (50 cavities in total). There was no statistically significance between the two groups according to age, gender or disease course (P>0.05); thus the groups were comparable for general information. This study was conducted in accordance with the Declaration of Helsinki and with approval from the Ethics Committee of the First Affiliated Hospital of Xinxiang Medical University (Weihui, China). Written informed consent was obtained from all participants.

### Treatment methods

Group A received a high dose of moxifloxacin over a shorter than usual treatment time. The patients in group A received moxifloxacin 0.6 g, once per day; amikacin, 0.6 g, once per day; pasiniazid, 1 g, once each night; pyrazinamide, 0.5 g, 3 times/day; rifabutin, 0.3 g, twice per day; pasiniazid, 0.3 g, 3 times/day and propylthiouracil isonicotinoyl amine, 0.2 g, 3 times/day, with a treatment course of 6 months. Patients in group B received normal treatment with moxifloxacin (0.4 g, once per day), in addition to the other antibtubercular drugs described above, as in group A, and the course of treatment was 9 months. Group A and group B received the same amount of moxifloxacin in total.

### Detection methods

Gollowing one course of treatment, monocytes and macrophages were isolated from the patients and cultured in Dulbecco’s modified Eagle’s medium (Gibco Life Technologies, Carlsbad, CA, USA) with 10% inactivated fetal bovine serum (Sijiqing, Hangzhou, China). at 37°C and 5% O_2_ for 1 week. Following the addition of 0.5 ml washing buffer to 1×10^6^ cells and centrifugation, another 1 ml washing buffer was added. Fluorescent goat anti-monkey immunoglobulin G-fluorescein isothiocyanate antibody (cat. no. FM38301; 1:16; Sigma-Aldrich, St. Louis, MO, USA) was then added to each tube. Following vibration of the tube, the changes in the expression of CD80, CD40 and HLA-DR on the cell surface were monitored by flow cytometry (EPICS^®^ ALTRA™; Beckman Coulter, Inc., Brea, CA, USA).

### Assessment of curative effect

The curative effect was assessed according to the guidelines approved by the Chinese Medical Association in 2005 (5). The rates of sputum negative conversion were determined at the end of 3, 6 and 9 months using the sputum smear test. A negative result means that no acid-fast bacilli were found in the sputum, and a positive result means that acid-fast bacilli were identified in the sputum. X-ray examination of lesions was also conducted. In terms of the proportion of lesions in the lung in the field of view: ‘evident absorption’ indicates resolution of ≥1/2 of the lesions; ‘absorption’ indicates resolution of ≥1/3 but <1/2 of the pathological changes; and ‘no change’ indicates <1/3 resolution of the pathological changes or an increase in pathological changes. In the evaluation of cavity improvement: ‘closure’ means scarless healing and the disappearance of obstruction; ‘shrinkage’ means narrowing of the cavity diameter by >1/2; ‘no change’ means narrowing of the cavity diameter by <1/2 or an increase in cavity diameter. In the comprehensive efficacy evaluation: ‘cured’ means no active tuberculosis in the lung and cavity closure; ‘markedly effective’ means sputum negative conversion, cavity reduction and the absorption of foci; ‘effective’ means sputum negative conversion, the absorption of or no change in foci, and reduction or no cavity changes; ‘invalid’ means sputum positive conversion, pathological changes and no cavity changes.

### Statistical analysis

The statistical software SPSS, version 17.0 (SPSS Inc, Chicago, IL, USA) was used to analyze the data. The data are expressed in numbers and percentages. The χ^2^ test was used to compare the differences between groups. P<0.05 was considered to indicate a statistically significant difference between two groups.

## Results

### Comparison of the curative effect between the two groups

Following treatment, the curative rate in group A was 82.61% and that in group B was 84.78%. There was no statistically significant difference in curative rate between the two groups (P>0.05; Table I).

### Comparison of the rates of sputum negative conversion at 3, 6 and 9 months between the two groups

**Figure 1:**
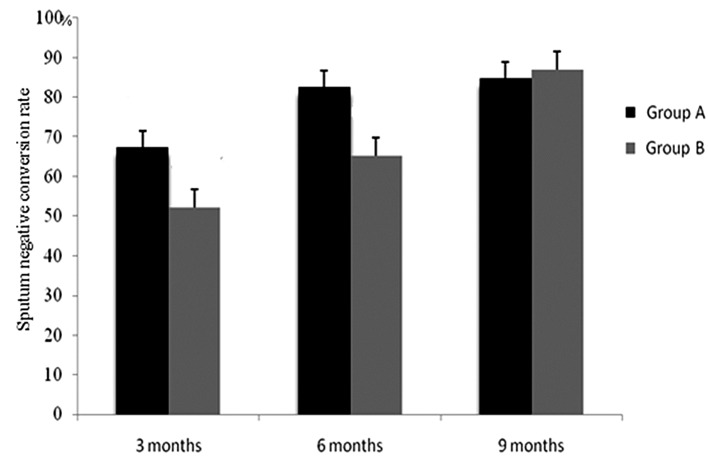
Comparison of the sputum negative conversion rates of the two groups after 3, 6 and 9 months of treatment.

Following 3 and 6 months of the treatment, the rates of sputum negative conversion in group A were significantly higher than those in group B, and there was observed to be a statistically significant difference between the two groups (χ_3_^2^=10.956, χ_6_^2^=13.565; P<0.01). However, there was no statistically significant difference in the rates of sputum negative conversion between the two groups at the end of the 9 months of treatment (χ_9_^2^=0.485; P>0.05; Fig. 1).

### Comparison of lesions and tuberculous cavities between the two groups following treatment

No statistically significant difference was identified in the improvement of lesions and cavity conditions between the two groups at the end of the treatment period (P>0.05; Table II).

### Comparison of adverse effects between the two groups

The main adverse effects observed in the two groups were reductions of peripheral white blood cell counts, liver function damage, gastrointestinal symptoms and neurological symptoms. The incidence of each of these adverse effects in group A was significantly lower than its incidence in group B (P<0.05; Table III).

### Effect of moxifloxacin on the expression of antigen-presenting functional molecules on the surfaces of mononuclear cells in the two groups

**Figure 2:**
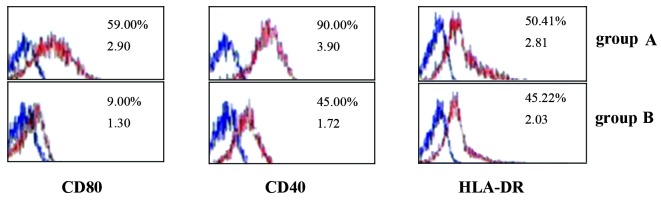
Effect of moxifloxacin on the expression of antigen-presenting functional molecules on mononuclear cell surfaces. The red line represents the expression level of mononuclear cytokines in the patients. The blue line represents the expression of normal cells. The percentages represent the expression of mononuclear cytokines and the numbers below indicate the ratio of the expression levels in patients to normal cells. P<0.05 for CD80 and CD40 expression in group A compared with group B; P>0.05 for HLA-DR expression in group A compared with group B.

In group A, on the surfaces of the mononuclear cells (monocytes and macrophages), the expression of antigen-presenting functional molecules CD80 and CD40 was higher than that in group B (P<0.05); however, the expression of HLA-DR was not significantly different between groups A and B (P>0.05; Fig. 2).

## Discussion

Fluoroquinolone antibiotics are critical in MDR-PTB treatment, and are known as second-line anti-tuberculosis drugs in the medical field. The mechanism of fluoroquinolone antibiotics is mainly the inhibition of the activity of DNA gyrase, and thus destruction of the replication and transcription of DNA in *Mycobacterium tuberculosis*, which further destroys the genetic material in the cells, leading to the death of *Mycobacterium tuberculosis* (6). However, as antibacterial drugs are widely used, the degree of drug-resistance of tuberculosis also increases. Therefore, the appropriate medication is vital for preventing an increase in the amount of MDR-PTB (7). Moxifloxacin is a relatively new 8-methoxyquinolone derivative, which has antibacterial activity against *Mycobacterium tuberculosis* and non-tuberculous mycobacteria (8). During the treatment of MDR-PTB, the duration of treatment with anti-*Mycobacterium tuberculosis* drugs is largely associated with sputum drug resistance; a longer duration of treatment usually leads to increasing drug-resistance and a worse curative effect (9). Therefore, the present study considered the condition of drug-resistance in patients with MDR-PTB. This study, with reference to tuberculosis prevention and control conducted in institutions in multiple regions, moderately increased the dose of moxifloxacin and decreased the length of the cycle of treatment, with the aim of decreasing the incidence of tolerance to moxifloxacin among patients.

The results indicated that there was no statistically difference in curative effect, sputum negative conversion rate, the resolution of lesions and cavity improvement between patients with MDR-PTB who received super-compact treatment with a high dose of moxifloxacin and those who received moxifloxacin treatment at the normal dosage and duration. However, the incidence of adverse effects in the patients who received super-compact treatment with a high dose of moxifloxacin was significantly reduced compared with that in the normal moxifloxacin treatment group. Abbate *et al* (10) observed that the antibacterial activity of moxifloxacin is 4–8-fold stronger than that of the antituberculosis drug levofloxacin and that patients with MDR-PTB are generally sensitive to it. Isoniazid may be used in combination with amantadine hydrochloride and *p-*aminosalicylic acid. Isoniazid mainly inhibits the proliferation and growth of mycobacteria. However, *p-*aminosalicylic acid slows down the acetylation of isoniazid in the organism, and provides a stable concentration of amantadine hydrochloride in the blood, which decreases the toxicity of isoniazid in the liver (11). Rifabutin is spiro derivative of piperidine and rifamycin; its activity against *Mycobacterium tuberculosis* activity is 4-fold stronger than that of the commonly clinically used drug rifampicin, and it is active against rifampicin-resistant tuberculosis (12,13). Amikacin, pasiniazid, pyrazinamide and propylthiouracil isonicotinoyl amine are classical anti-tuberculosis drugs. In the present study, the advantages of the new moxifloxacin administration strategy were demonstrated to be high activity against *Mycobacterium tuberculosis*, with evident bactericidal activity and applicability for patients with tuberculosis resistant to multiple drugs, including rifampin, ofloxacin and rifampicin. In addition, this new strategy was found to be safe, with high tolerance, which supports the proposition of super-compact treatment with a high dose of moxifloxacin for MDR-PTB (14). In addition, Liang *et al* (15) found that long-term anti-tuberculosis treatment is highly likely to be associated with new infections, and the pulmonary lesions were mainly exudative. This explains to some extent why fewer adverse effects occur when anti-tuberculosis treatment is conducted for a shorter time period. The limitation of the present study is that the cost of treatment with moxifloxacin and rifabutin is high. Thus, it would be difficult to clinically apply this new treatment strategy; this safer strategy is only likely to be available to patients who have good economic conditions.

Mononuclear macrophages are antigen-presenting cells, with large quantities of antigen-presenting functional molecules on the surface. Mononuclear macrophages may present tuberculosis antigen to T cells (CD4^+^ and CD8^+^), activate the immune response to differing degrees, and induce the accumulation of T cells. Therefore, antigen-presenting functional molecules on mononuclear cell surfaces have important immunological functions against tuberculosis. In the present study, in group A, the expression of the antigen-presenting functional molecules CD80 and CD40 on the mononuclear cell surface was greater than that in group B, but no significant difference in HLA-DR expression was observed between the two groups. This indicates that moxifloxacin treatment over a shorter time period may induce the release of many cytokines, facilitate activation of the immune response and increase the immune function in patients with tuberculosis. However, these effects decreased gradually during the long-term treatment with moxifloxacin. The study only compared moxifloxacin treatments of different concentrations and durations, without an empty control, and only focused on the mechanism by which moxifloxacin affects T cells; an investigation of the overall mechanism is lacking. Therefore, further research and investigation are needed.

In summary, the short-duration treatment with a high dose of moxifloxacin is effective for MDR-PTB, and its advantages are a reduction in the incidence of adverse reactions and a lack of drug resistance. The treatment strategy used in the present study is worthy of further study and testing in clinical trials.

## Figures and Tables

**Table I:** Comparison of curative effect between the two groups.

		Efficacy, n				
			
Group	No. of cases	Cured	Markedly effective	Effective	Invalid	Curative rate (%)
A	46	5	19	14	8	82.61
B	46	4	17	18	7	84.78
χ^2^						0.526
P-value						>0.05

**Table II:** Comparison of lesions and tuberculous cavities between the two groups after treatment.

	Lesions, n (%)				Cavities, n (%)			
		
Group	No. of cases	Evident absorption	Absorption	No change	Total no.	Closure	Shrinkage	No change
A	46	14 (30.44)	26 (56.52)	6 (13.04)	48	31 (64.58)	7 (14.58)	10 (20.83)
B	46	12 (26.09)	27 (58.70)	7 (15.22)	50	31 (62.00)	8 (16.00)	11 (22.00)
χ^2^		2.145	1.236	1.321		1.452	1.756	1.358
P-value		>0.05	>0.05	>0.05		>0.05	>0.05	>0.05

**Table III:** Comparison of adverse effects between the two groups [n (%)].

Group	No. of cases	Reduction of white blood cell count	Liver function damage	Gastrointestinal symptoms	Neurological symptoms
A	46	1 (2.17)	2 (4.35)	5 (10.87)	6 (13.04)
B	46	5 (10.87)	5 (10.87)	14 (30.45)	12 (26.09)
χ^2^		4.125	3.892	16.125	8.751
P-value		<0.05	<0.05	<0.01	<0.01
